# Rare Orbital Metastasis Originating from Ampullary Adenocarcinoma

**DOI:** 10.3390/medicina57111238

**Published:** 2021-11-12

**Authors:** Yung-En Tsai, Ke-Hung Chien, Yao-Feng Li, Shiue-Wei Lai

**Affiliations:** 1 Department of Ophthalmology, Kaohsiung Armed Forces General Hospital, Kaohsiung 802, Taiwan; zun7511@gmail.com; 2 Department of Ophthalmology, Tri-Service General Hospital, National Defense Medical Center, Taipei 114, Taiwan; yred8530@gmail.com; 3 Department of Pathology, Tri-Service General Hospital, National Defense Medical Center, Taipei 114, Taiwan; liyaofeng1109@gmail.com; 4 Division of Hematology and Oncology, Department of Internal Medicine, Tri-Service General Hospital, National Defense Medical Center, Taipei 114, Taiwan

**Keywords:** orbital tumor, orbital metastasis, ampullary carcinoma

## Abstract

Background: Orbital metastasis from ampullary carcinoma is rare, with no previously reported cases. Case presentation: We report the case of a 60-year-old man who complained of a right-sided headache, blurred vision, progressive proptosis, ptosis, and right eye pain for 3 months. His past medical history included an ampullary adenocarcinoma stage IIIA treated via the Whipple procedure and adjuvant chemoradiotherapy 1 year ago. However, he was lost to follow-up. Computed tomography of the orbit showed a soft tissue lesion in the right orbital fossa measuring 3.3 × 2 × 2 cm. An orbital mass biopsy demonstrated an intestinal-type adenocarcinoma that tested positive for cytokeratins 7 and 20 and CDX2 on immunohistochemical staining. The pathologic diagnosis was metastatic adenocarcinoma from the ampulla of Vater. Despite oncological treatment, the patient’s illness progressed. He received palliative treatment and died 1 month later. Conclusions: We presented a rare case of orbital metastasis from ampullary adenocarcinoma. This should be considered in the differential diagnosis of patients with a history of ampullary adenocarcinoma who present with symptoms referring to the relevant locations.

## 1. Introduction

Metastatic orbital tumors, especially from primary ampullary carcinomas, are rare. The most common primary cancers with orbital metastases are breast, lung, and prostate cancers. Ampullary carcinoma is also rare, which arises from the ampullary complex distal to the confluence of the common bile and pancreatic duct and typically metastasizes to the regional nodes, liver, adjacent organs, and lungs. Other less common metastasis sites include the brain and skeletal system [1]. Orbital metastasis from ampullary carcinoma is rare, and to the best of our knowledge there have been no reports on this entity in the English literature. We report a rare case of orbital metastasis from ampullary adenocarcinoma.

## 2. Case Report

A 60-year-old man presented with a right-sided headache, blurred vision, progressive proptosis, ptosis, and pain in the right eye noted 3 months ago. He reported a 10 kg weight loss within the past 6 months. The patient has smoked 1.5 packs of cigarettes per day and consumed alcohol for more than 40 years. His past medical history included an ampullary adenocarcinoma stage IIIA treated with the Whipple procedure followed by chemoradiotherapy 1 year ago. He was lost to follow-up after one cycle of chemoradiotherapy.

**Figure 1:**
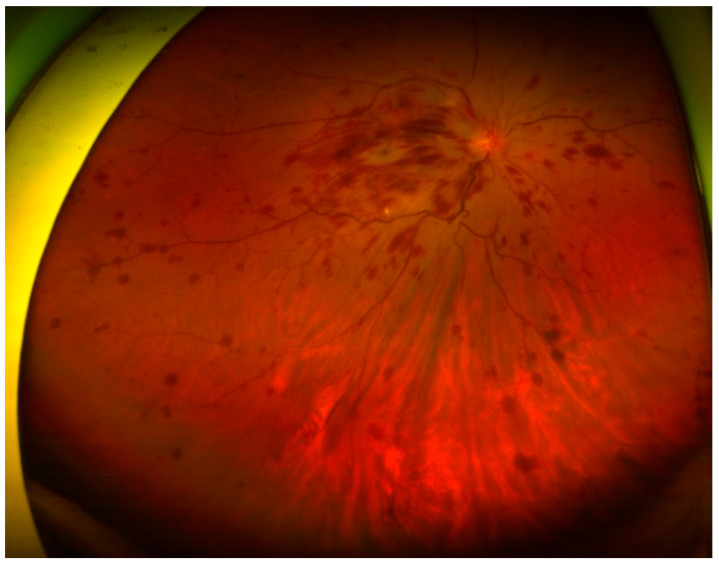
Funduscopic examination showing papilledema and flame-shaped hemorrhage over the four quadrants of the right eye.

**Figure 2:**
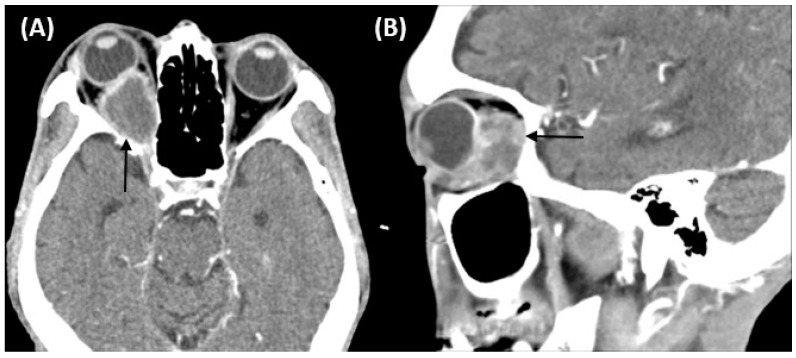
Computed tomography of the orbit demonstrating a soft tissue lesion measuring 3.3 × 2 × 2 cm in the right orbital fossa with peripheral enhancement (black arrow). The right globe and optic nerve are compressed, causing exophthalmos. (**A**) Axial image. (**B**) Sagittal image.

**Figure 3:**
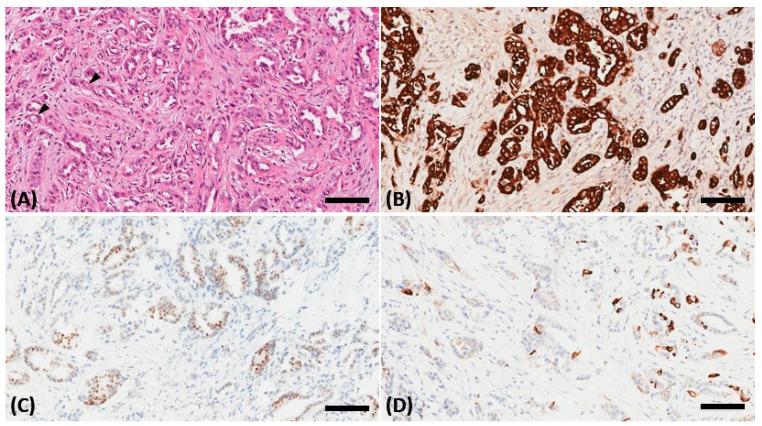
A biopsy of the orbital mass revealing an intestinal-type adenocarcinoma in the hematoxylin and eosin stain characterized by a glandular tumor growth pattern with scattered goblet cell differentiation (black arrowhead) and hyperchromatic nuclei (**A**). On immunohistochemical staining, the tumor cells showed positive staining for cytokeratin (CK)7 (**B**), scatter staining for CK20 (**C**), and focal nuclear staining for CDX2 (**D**). The scale bar is 100 μm.

Ophthalmologic examination revealed an intraocular pressure of 26.5 mm Hg in the right eye and 10 mm Hg in the left eye with proptosis and limited right eye movement in all directions. The left eye movements in all directions were intact. Upon examining visual acuity, the right eye exhibited light perception while the left eye had an acuity of 20/20. The right pupil was fixed and mid-dilated with no light reflex. A slit-lamp examination revealed dense bilateral cataracts. A funduscopic examination showed papilledema and flame-shaped hemorrhage over the four quadrants of the right eye (Figure 1). Computed tomography of the orbit demonstrated a soft tissue lesion measuring 3.3 × 2 × 2 cm in the right orbital fossa with peripheral enhancement. The right globe and optic nerve were compressed, causing exophthalmos (Figure 2). A biopsy of the orbital mass revealed an intestinal-type adenocarcinoma characterized by a glandular tumor growth pattern with scattered goblet cell differentiation and hyperchromatic nuclei. On immunohistochemical staining, the tumor cells had positive staining for cytokeratin (CK)7, scatter staining for CK20, and focal nuclear staining for CDX2 (Figure 3). The pathologic diagnosis was metastatic adenocarcinoma from the ampulla of Vater. Multiple metastases were noted after complete examination and imaging. After one cycle of chemotherapy, consisting of high-dose 5-fluorouracil (5-FU), his illness progressed and was complicated by secondary infection with sepsis. Refractory hypoglycemia was observed despite intravenous glucose administration. Further evaluation showed a decreased serum insulin-like growth factor-1 (IGF1) at 15 ng/mL (reference range, 81–225 ng/mL). Additionally, the serum insulin (<0.5 mU/L) and C-peptide (<0.05 ng/mL) were suppressed. Paraneoplastic syndrome of non-islet cell tumor hypoglycemia (NICTH) was also considered. The patient received palliative treatment and died 1 month later.

## 3. Discussion

Orbital metastases account for 1–13% of orbital tumors [2]. The prevalence of orbital metastasis in patients with cancer ranges from 2% to 4.7% [2]. The leading causes of orbital metastases include breast, lung, and prostate carcinomas, as well as cutaneous melanomas [2]. Orbital metastasis from primary ampullary carcinoma is rare, with no reported cases. To the best of our knowledge, this is the first report of orbital metastasis from an ampullary adenocarcinoma.

Ampullary carcinoma is a rare tumor, accounting for approximately 0.2% of gastrointestinal malignancies and 7% of periampullary cancers [3]. Most ampullary carcinomas are adenocarcinomas, but their histologic tumor variants include papillary, adenosquamous, mucinous, and adenocarcinomas [3]. The ampulla of Vater represents the epithelial junction of the main pancreatic duct and the distal bile duct surrounded by the parenchyma of the pancreatic head and duodenum. Ampullary carcinoma can arise from the intestinal epithelium or the epithelium covering the pancreaticobiliary ducts [4]. Ampullary adenocarcinoma consists of two main histological subtypes: intestinal and pancreaticobiliary. These histological subtypes have different clinical characteristics, and defining the exact histological type is essential for prognostication. The intestinal-type ampullary adenocarcinoma has a more favorable prognosis than the pancreaticobiliary type (median overall survival of 115.5 vs. 16 months; *p* < 0.001, respectively) [5]. The incidence of ampullary carcinoma peaks during the seventh and eighth decades of life. Men are more commonly affected than women, with a reported male-to-female ratio of 1.48:1 [6]. Risk factors for ampullary carcinoma development include old age, male sex, and hereditary polyposis syndromes, such as familial adenomatous polyposis and Lynch syndrome [7].

Approximately 50% of patients with ampullary carcinoma present at an advanced stage [8]. Chemotherapy is crucial in treating ampullary carcinoma, especially in patients with distant metastasis, recurrence, or unresectable locally advanced disease [9]. In this study, the patient’s condition deteriorated after receiving one cycle of chemotherapy with a high-dose 5-FU; palliative care was recommended by multidisciplinary tumor conferences.

Additionally, our patient presented with refractory hypoglycemia despite receiving intravenous glucose. Paraneoplastic syndrome of NICTH was considered. NICTH is a rare disease caused by tumor-secreting substances that induce hypoglycemia via a non-insulin-mediated mechanism. NICTH presents as recurrent or constant hypoglycemic episodes and most commonly affects elderly patients with advanced tumors. NICTH is characterized by low serum insulin, C-peptide, growth hormone, and IGF1 levels; normal or elevated IGF2 levels; and a high IGF2:IGF1 ratio [10].

In conclusion, we present a rare case of orbital metastasis from ampullary adenocarcinoma. This should be considered in the differential diagnosis of patients with a history of ampullary adenocarcinoma who present with symptoms referring to the relevant locations.

## Data Availability

Not applicable.
